# Hyperkalemia and Risk of CKD Progression: A Propensity Score–Matched Analysis

**DOI:** 10.34067/KID.0000000000000541

**Published:** 2024-08-09

**Authors:** Abiy Agiro, Erin Cook, Fan Mu, Alexandra Greatsinger, Jingyi Chen, Angela Zhao, Elaine Louden, Ellen Colman, Pooja Desai, Glenn M. Chertow

**Affiliations:** 1 AstraZeneca, Wilmington, Delaware; 2 Analysis Group, Inc., Boston, Massachusetts; 3 Analysis Group, Inc., New York, New York; 4 Stanford University School of Medicine, Stanford, California

**Keywords:** CKD, clinical epidemiology, epidemiology and outcomes, kidney disease, progression, renal progression

## Abstract

**Key Points:**

- Hyperkalemia is a known complication of CKD; however, it is not known whether hyperkalemia directly contributes to CKD progression and risk of death.
- We found that patients with stages 3b/4 CKD and hyperkalemia had higher risk of CKD progression and death than matched patients without hyperkalemia.

**Background:**

Hyperkalemia is a known complication of CKD; however, it is not known whether hyperkalemia directly contributes to CKD progression and the risk of death. Clarifying the extent to which hyperkalemia is associated with CKD progression and mortality can inform clinical practice and guide future research. The objective of this study was to quantify the risks of CKD progression and mortality associated with hyperkalemia in patients with stages 3b/4 CKD.

**Methods:**

This was a real-world, exact and propensity score matched, observational cohort study using data (January 2016 to December 2021) from Optum's deidentified Market Clarity Data, a large US integrated insurance claims/electronic medical record database. The study included matched adult patients with stages 3b/4 CKD with and without hyperkalemia, not regularly treated with an intestinal potassium (K^+^) binder. Measured outcomes were CKD progression and all-cause mortality. CKD progression was defined as diagnosis of CKD stage 4 (if stage 3b at index), CKD stage 5 or kidney failure, or receipt of dialysis or kidney transplantation.

**Results:**

After matching, there were 6619 patients in each of the hyperkalemia and nonhyperkalemia cohorts, with a mean follow-up time of 2.12 (SD, 1.42) years. Use of any renin-angiotensin-aldosterone system inhibitors during baseline was common (75.9%), and most patients had CKD stage 3b (71.2%). Patients with hyperkalemia had a 1.60-fold (95% confidence interval, 1.50 to 1.71) higher risk of CKD progression and a 1.09-fold (1.02 to 1.16) higher risk of all-cause mortality relative to patients without hyperkalemia. Relative risks of CKD progression associated with hyperkalemia were similar within the subset of patients receiving renin-angiotensin-aldosterone system inhibitor, across CKD stages, and when alternative definitions of CKD progression were used.

**Conclusions:**

Patients with CKD stages 3b/4 and hyperkalemia experienced significantly higher risks of CKD progression and all-cause mortality than propensity score matched patients without hyperkalemia.

## Visual Abstract

**Figure:**
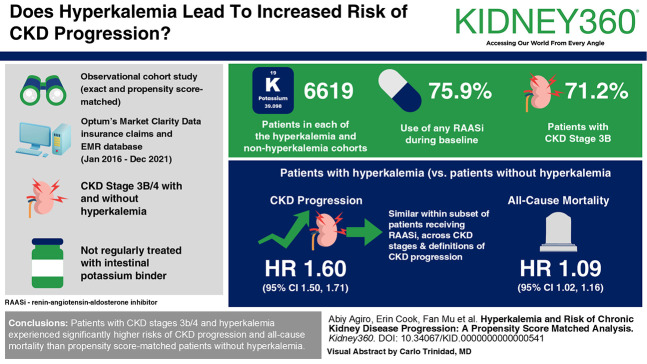

## Introduction

Hyperkalemia is the elevation of serum potassium (K^+^) concentrations (K^+^ >5.0 mmol/L) often caused by impaired kidney function.^1^ Hyperkalemia is a common complication of CKD or AKI.^2^ Risk factors of hyperkalemia in patients with CKD include advanced disease stage; comorbidities, including diabetes mellitus, heart disease, stroke, and adrenal disease; and use of renin-angiotensin-aldosterone system inhibitors (RAASi; *e.g*., angiotensin-converting enzyme inhibitors, angiotensin receptor blockers, and mineralocorticoid receptor antagonists) that are used to slow disease progression.^3^ The occurrence of hyperkalemia often prompts discontinuation of RAASi therapy,^4–6^ which can promote CKD progression to kidney failure and increase the risks of cardiovascular events and mortality.^7–9^ Thus, a challenge in CKD management is maximizing the benefits of RAASi therapy while mitigating hyperkalemia.^10^

The association between hyperkalemia and outcomes in CKD has been suggested in previous research.^4,5,11–26^ Serum K^+^ concentrations >5.0 mmol/L were shown to be associated with a 60% increased risk of kidney failure compared with the reference range of 4.5–5.0 mmol/L in patients with CKD stages G1–G4.^26^ Serum K^+^ concentrations >5.0 mmol/L were also associated with a decline in residual kidney function and up to a 56% higher risk of mortality in patients receiving maintenance dialysis compared with patients with serum K^+^ 4.0–4.5 mmol/L.^11^ These associations between hyperkalemia and outcomes are supported by the results of a meta-analysis of 27 international cohorts in the CKD Prognosis Consortium showing that moderate-to-severe hyperkalemia (K^+^ >5.5 mmol/L) was associated with higher risks of progression to kidney failure by 44% and all-cause mortality by 22% compared with a serum K^+^ concentration of 4.2 mmol/L.^21^ However, the contribution of hyperkalemia to CKD progression before kidney failure is unclear.

Clarifying the extent to which hyperkalemia increases the risk of CKD progression and mortality can inform clinical management strategies, including the use of intestinal K^+^ binders that increase K^+^ excretion.^27–30^ To this end, this study sought to quantify the risks of CKD progression and mortality associated with hyperkalemia by examining data in a carefully characterized matched cohort of patients with CKD stages 3b/4 with and without hyperkalemia.

## Methods

### Study Design

This observational study, Revolutionize II, was a real-world matched cohort study of patients with CKD stages 3b/4 evaluating the association between any hyperkalemia (≥1 event of hyperkalemia) and long-term outcomes. We used data (January 2016 to December 2021) from Optum's deidentified Market Clarity Data, a large US integrated insurance claims/electronic medical record database. The index date for the hyperkalemia cohort was the date of first CKD stage 3/4 diagnosis that came after a diagnosis of hyperkalemia, and the index date for the nonhyperkalemia cohort was a randomly selected CKD stages 3/4 diagnosis date. There were no International Classification of Diseases, 10th revision diagnosis codes for substages of CKD at the time of the study, so CKD substage was confirmed using a combination of International Classification of Diseases diagnosis codes for CKD stage 3 or stage 4 and eGFR values indicating CKD substages.

The baseline period was the 12 months preindex, and the follow-up period spanned from the index date to the earliest among end of continuous enrollment, end of data availability, outpatient intestinal K^+^ binder use for ≥1 week, or death.

The data were deidentified and comply with requirements of the Health Insurance Portability and Accountability Act of 1996; therefore, no review by an Institutional Review Board was required.

### Study Population

**Figure 1:**
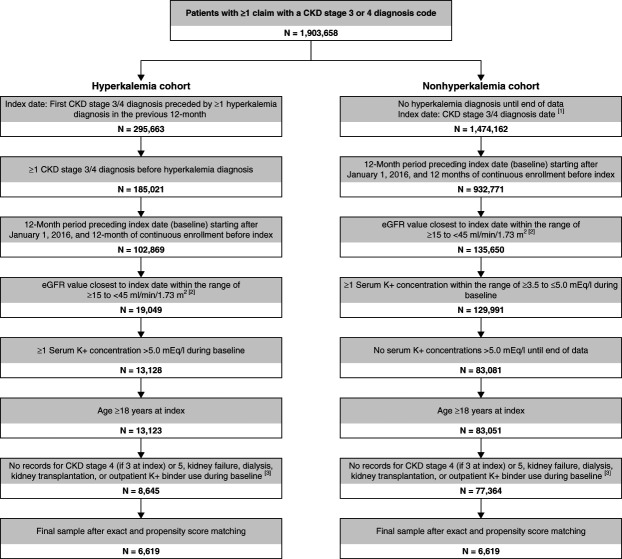
**Selection of patients with hyperkalemia not treated with potassium binders (hyperkalemia cohort) and those without hyperkalemia (nonhyperkalemia cohort).** [1] Patients could have multiple index dates. [2] eGFR was assessed in the outpatient setting only, whereas serum K^+^ was assessed in any setting. [3] Patients with advanced CKD stages (*i.e*., 4 [if stage 3 at index], 5, or kidney failure) during baseline were excluded based on diagnosis codes. K^+^, potassium.

The overall study population comprised of matched adult patients with CKD, with or without hyperkalemia; a patient selection diagram is shown in Figure 1. 

#### Inclusion and Exclusion Criteria

Inclusion criteria for the hyperkalemia cohort were as follows: ≥1 claim with a CKD stages 3/4 diagnosis code preceded by a hyperkalemia diagnosis in the baseline period; ≥1 claim with a CKD stages 3/4 diagnosis code preceding the hyperkalemia diagnosis associated with the index date (to ensure that patients had CKD at the time of hyperkalemia diagnosis); and ≥1 serum K^+^ concentration >5.0 mmol/L in any setting (inpatient or outpatient) during the baseline period.

Inclusion criteria for the nonhyperkalemia cohort were as follows: ≥1 claim with a CKD stages 3/4 diagnosis code (randomly selected from potential index dates that met the inclusion and exclusion criteria); no history of hyperkalemia (*i.e*., no hyperkalemia diagnoses or serum K^+^ >5.0 mmol/L before or after index); and ≥1 serum K^+^ concentration ≥3.5 to ≤5.0 mmol/L in any setting in the baseline period.

All patients were required to meet the following criteria: ≥12 months of continuous medical and pharmacy enrollment preindex, outpatient eGFR value closest to the index date of ≥15 to <45 ml/min per 1.73 m^2^, and age ≥18 years at the index date.

We excluded patients from both cohorts if they had ≥1 claim for an outpatient intestinal K^+^ binder, kidney transplant, dialysis, or a diagnosis code for CKD stage 4 (if stage 3b at index), stage 5, or kidney failure during baseline.

#### Matching

We matched patients 1:1 with and without hyperkalemia using a two-step process of exact and propensity score matching to control for confounding in these variables by ensuring that the distribution of key patient characteristics was the same between the hyperkalemia and normokalemia cohorts. First, we exactly matched patients with and without hyperkalemia on age (<75, ≥75 years), Medicare insurance, CKD stage (diagnosis code), eGFR (<30, 30 to <45, ≥45 ml/min per 1.73 m^2^), RAASi use, a composite measure of hospitalization for myocardial infarction or stroke, and a composite measure of hospitalization for myocardial, stroke, or heart failure.

Next, patients were propensity score matched within each coarsened exact match category using caliper matching with a caliper of 0.01. We generated the propensity score using a logistic regression model predicting hyperkalemia based on baseline characteristics. Variables related to hyperkalemia included in the model were determined based on the literature^31^ and clinical input, with additional covariates selected from a large number of candidate variables using a previously developed algorithm.^32^ The following covariates were included in the final model: age at index (continuous), sex, designated race or ethnicity, geographic region, insurance type, eGFR at index (continuous), AKI-related hospitalization, variables used for exact matching, and baseline clinical characteristics selected using a ranking algorithm—namely, comorbidities (*i.e*., coronary artery disease, type 1 diabetes, type 2 diabetes, AKI, edema, heart failure, mild liver disease, diabetes without complications, diabetes with complications, and renal disease), and use of *β* blockers.

Patients were matched 1:1 within each coarsened exact match category using the propensity score and caliper matching. This two-step process is intended to remove the potential risk of confounders by observed characteristics by ensuring the distribution of these characteristics is similar between cohorts.^33,34^

#### Subanalyses

We performed analyses initially in the overall population and later in prespecified subanalyses. Subanalyses included patients receiving RAASi during baseline, baseline CKD stage (defined using diagnosis codes), type 2 diabetes, recurrent hyperkalemia, and mild baseline hyperkalemia (serum K^+^ >5.0 and ≤5.5 mmol/L using value closest to index). Additional details on the subanalyses tested are included in the Supplemental Methods and the selection of patients with recurrent hyperkalemia is illustrated in Supplemental Figure 1.

### Outcomes

Outcomes evaluated in this study were CKD progression (primary end point) and all-cause mortality (secondary end point). CKD progression was defined as diagnosis code of CKD stage 4 (if stage 3b at index), CKD stage 5, or kidney failure (defined as receipt of dialysis or kidney transplantation). The primary definition of CKD progression served as the primary end point. A secondary definition of CKD progression was a diagnosis code of CKD stage 5 or kidney failure (excluding transition from CKD stage 3b to stage 4). The exploratory definition of CKD progression was a 30% decline in eGFR from baseline ≥4 weeks after index or receipt of dialysis or kidney transplantation, assessed in a subset of patients with ≥1 eGFR laboratory value recorded during baseline and ≥1 eGFR laboratory value recorded ≥4 weeks after index.

### Variables

We assessed patient and clinical characteristics, including demographics, comorbidities, medication use, and health care resource utilization during baseline, in the hyperkalemia and nonhyperkalemia cohorts before and after matching. We assessed all outcomes in the overall population during follow-up and primary CKD progression and all-cause mortality in the prespecified subanalyses during follow-up.

### Statistical Analysis

We summarized baseline characteristics using means, SDs, medians, and 25%–75% ranges for continuous variables and counts and proportions for categorical variables. We compared patient characteristics between matched cohorts using standardized mean differences. We considered characteristics with standardized mean difference <0.200 to be well matched.

We evaluated time to CKD progression using Kaplan–Meier product limit estimates. We used adjusted cause-specific Cox proportional hazard regression models to compare the risk of CKD progression between matched cohorts, accounting for death as a competing risk, and the risk of death. We used robust sandwich variance estimators to account for correlations between matched pairs. We considered 2-tailed *P* values < 0.05 statistically significant. We conducted all analyses using R 3.6.3.

## Results

### Characteristics of the Study Population

#### Demographics and Clinical Characteristics

A total of 86,009 patients (8645 with hyperkalemia and 77,364 without hyperkalemia) met the inclusion criteria (Figure 1). After matching, there were 6619 patients each in the hyperkalemia and nonhyperkalemia cohorts, with a mean follow-up time of 2.12 (SD, 1.42) years (median: 2.05 [25%–75% range 0.85–3.24]). Baseline characteristics of the matched cohorts are presented in Table 1 and were similar among patients with and without hyperkalemia, as expected. In the overall population, the mean age was 74.5 (SD, 11.3) years (median: 77.1 years [25%–75% range 67.9–84.2 years]), and a larger proportion of the sample was female (53.0%). Based on outpatient eGFR value closest to the index, the majority of patients had CKD stage 3b (71.2%). In the hyperkalemia cohort, patients had a mean of 3.4 (SD, 2.0) hyperkalemia events detected (diagnoses or serum K^+^ concentrations >5.0 mmol/L) (median 3.0 [25%–75% range 2.0–4.0]), with a mean highest K^+^ concentration during baseline of 5.6 (SD, 0.4) mmol/L (median 5.5 mmol/L [25%–75% range 5.3–5.8 mmol/L]). The prevalence of hyperkalemia-related and cardiovascular-related comorbidities was similar between the two cohorts. The most common hyperkalemia-related comorbidities were hypertension (hyperkalemia, 92.6%; nonhyperkalemia, 92.2%) and type 2 diabetes mellitus (60.1%; 60.4%, respectively), and the most common cardiovascular-related comorbidities were heart failure (hyperkalemia, 37.6%; nonhyperkalemia, 37.4%) and peripheral vascular disease (34.5%; 31.6%, respectively). Baseline medication use and the proportion of patients with inpatient stays or emergency department visits were similar between the two groups, although a higher proportion of patients in the hyperkalemia cohort received angiotensin-converting enzyme inhibitors and mineralocorticoid receptor antagonists at baseline, while a higher proportion of patients in the nonhyperkalemia cohort received angiotensin receptor blockers at baseline (Table 1).

**Table 1:** Baseline characteristics of the study population

Characteristic	Cohort		SMD^a^
	Hyperkalemia (*n*=6619)	Nonhyperkalemia (*n*=6619)	
**Demographics**			
Age, yr	74.5±11.3	74.5±11.4	0.001
Female	3508 (53.0)	3508 (53.0)	0.000
Race			0.011
*Asian*	106 (1.6)	103 (1.6)	
*Black*	972 (14.7)	992 (15.0)	
*Other/unknown*	513 (7.8)	501 (7.6)	
*White*	5028 (76.0)	5023 (75.9)	
Medical insurance type			0.054
*Commercial*	1240 (18.7)	1240 (18.7)	
*Medicaid*	386 (5.8)	442 (6.7)	
*Medicare*	4699 (71.0)	4699 (71.0)	
*Unknown*	294 (4.4)	238 (3.6)	
**Clinical characteristics**			
BMI, kg/m^2^	30.8±7.6	31.5±7.6	0.086
CKD stage			
*Diagnosis code at index*			0.000
Stage 3	4912 (74.2)	4912 (74.2)	
Stage 4	1707 (25.8)	1707 (25.8)	
*eGFR closest to index^b^, ml/min per 1.73 m*^*2*^			0.000
Stage 3b (30 to <45)	4715 (71.2)	4715 (71.2)	
Stage 4 (15 to <30)	1904 (28.8)	1904 (28.8)	
Mean±SD	34.0±7.4	34.1±7.5	0.008
Median (25%–75% range)	35.1 (28.9–40.2)	35.4 (28.7–40.4)	
Hyperkalemia			
*Total number of hyperkalemia events during baseline*			
Mean±SD	3.4±2.0	0.0±0.0	2.405
Median (25%–75% range)	3.0 (2.0–4.0)	0.0 (0.0–0.0)	
Serum K^+^ concentration closest to index date, mmol/L			
*Mean±SD*	5.0±0.6	4.2±0.4	1.627
*Median (25%–75% range)*	5.1 (4.7–5.4)	4.2 (3.9–4.5)	
*Hyperkalemia severity*			1.558
≤5	2991 (45.2)	6619 (100.0)	
>5 to <5.5	2181 (33.0)	0 (0.0)	
5.5 to <6	1180 (17.8)	0 (0.0)	
≥6	267 (4.0)	0 (0.0)	
Most severe K^+^ concentration during baseline, mmol/L			
*Mean±SD*	5.6±0.4	4.4±0.4	3.071
*Median (25%–75% range)*	5.5 (5.3–5.8)	4.5 (4.2–4.7)	
*Hyperkalemia severity*			0.873
≤5	0 (0.0)	6619 (100.0)	
>5 to <5.5	3009 (45.5)	0 (0.0)	
5.5 to <6	2765 (41.8)	0 (0.0)	
≥6	845 (12.8)	0 (0.0)	
Comorbidities			
*Charlson Comorbidity Index*	3.6±2.2	3.6±2.2	0.004
*Hyperkalemia-related comorbidities*			
AKI	2627 (39.7)	2601 (39.3)	0.008
Coronary artery disease	2652 (40.1)	2687 (40.6)	0.011
Type 2 diabetes	3975 (60.1)	3997 (60.4)	0.007
Hypertension	6126 (92.6)	6103 (92.2)	0.013
Edema	2084 (31.5)	2069 (31.3)	0.005
*Cardiovascular-related comorbidities*			
Cerebrovascular disease	1361 (20.6)	1434 (21.7)	0.027
Congestive heart failure	2487 (37.6)	2478 (37.4)	0.003
Myocardial infarction	912 (13.8)	911 (13.8)	0.000
Peripheral vascular disease	2282 (34.5)	2089 (31.6)	0.062
**Medication use**			
Any RAASi	5022 (75.9)	5022 (75.9)	0.000
*ACE inhibitors*	2978 (45.0)	2505 (37.8)	0.145
*ARB*	2017 (30.5)	2464 (37.2)	0.143
*ARN inhibitors*	124 (1.9)	93 (1.4)	0.037
*MRA*	1098 (16.6)	698 (10.5)	0.177
*β* blockers	4036 (61.0)	4102 (62.0)	0.020
**Health care resource utilization**			
Any inpatient visits	2187 (33.0)	2088 (31.5)	0.032
*Any CKD-related inpatient visit*	1862 (28.1)	1703 (25.7)	0.054
Any emergency department visits	1908 (28.8)	1877 (28.4)	0.010

Values are reported as mean±SD, median (25%–75% range), or *n* (%). ACE, angiotensin-converting enzyme; ARB, angiotensin receptor blocker; ARN, angiotensin receptor/neprilysin; BMI, body mass index; K^+^, serum potassium concentration; MRA, mineralocorticoid receptor antagonist; RAASi, renin-angiotensin-aldosterone system inhibitor; SMD, standardized mean difference.

a Standardized mean difference values >0.2 indicate an imbalance between cohorts.

b eGFR was assessed in the outpatient setting

#### Subanalyses

The number of matched pairs in in the subanalyses (RAASi; CKD stage; mild hyperkalemia) ranged from 1707 to 5022 (Supplemental Figure 2). Characteristics of the various subanalyses are shown in Supplemental Tables 1–7.

### Hyperkalemia and CKD Progression

**Figure 2:**
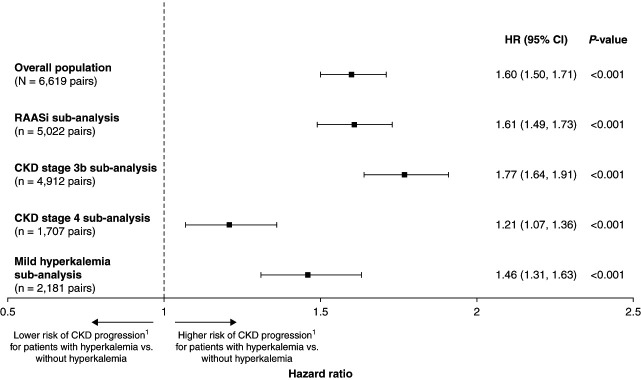
**Forest plot of CKD progression HR in the overall patient population and key subanalyses.** ^1^CKD progression was defined as a diagnosis of CKD stage 4 (if stage 3 at index) or 5, kidney failure, dialysis, or kidney transplantation. CI, confidence interval; HR, hazard ratio; RAASi, renin-angiotensin-aldosterone system inhibitor.

**Figure 3:**
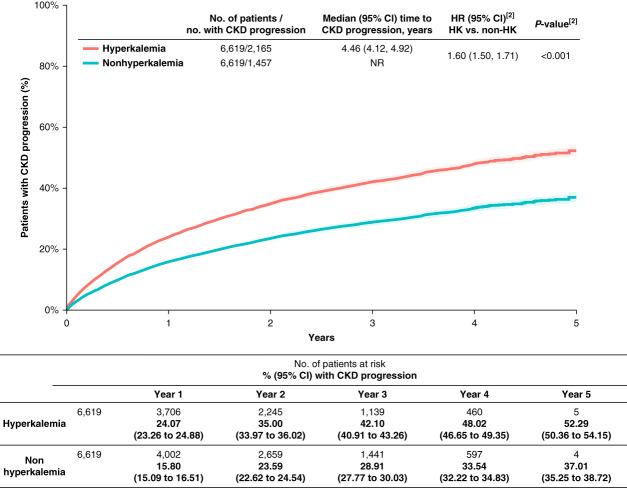
**Time to CKD progression in the overall patient population.**^**[1]**^ [1] CKD progression was defined as a diagnosis of CKD stage 4 (if stage 3 at index) or 5, kidney failure, dialysis, or kidney transplantation. [2] HR and *P* value were calculated from a cause-specific Cox proportional hazard model. The cluster function was used in the model formula to account for correlations between matched pairs and specify the use of robust sandwich variance estimators. HK, hyperkalemia; non-HK, non-hyperkalemia; NR, not reached.

Patients with hyperkalemia not prescribed regular outpatient intestinal K^+^ binders experienced a 1.60-fold higher risk of CKD progression compared with patients without hyperkalemia (95% confidence interval [CI], 1.50 to 1.71; *P* < 0.001). A total of 2165 patients with hyperkalemia and 1457 patients without hyperkalemia experienced CKD progression during follow-up. The results were consistent in the RAASi, CKD stage, and mild hyperkalemia subanalyses, with hazard ratios ranging from 1.21 to 1.77 (all *P* < 0.001; Figure 2). The median time (95% CI) to CKD progression was 4.46 (4.12 to 4.92) years for the hyperkalemia cohort and not reached for the nonhyperkalemia cohort (Figure 3). Similar and statistically significant results were obtained in the additional subanalyses (Supplemental Figure 3) and with the secondary and exploratory definitions of CKD progression (Supplemental Figure 4). For example, patients with hyperkalemia had a 1.88-fold higher risk of CKD progression (exploratory definition: 30% decline in eGFR from baseline at least 4 weeks after the index date or a procedure code for dialysis or kidney transplant) (95% CI, 1.68 to 2.09; *P* < 0.001) than patients without hyperkalemia.

### Hyperkalemia and All-Cause Mortality

**Figure 4:**
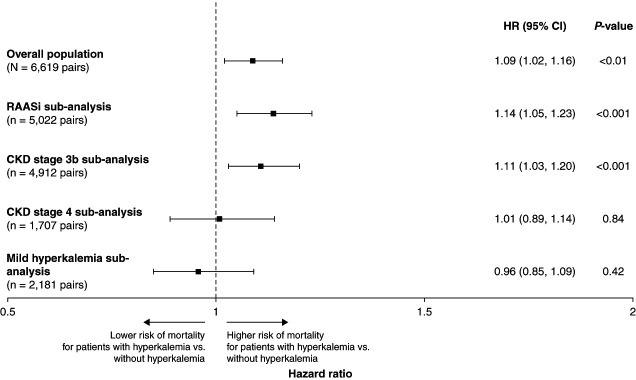
Forest plot of mortality HR in the overall patient population and key subanalyses.

The risk of all-cause mortality was 1.09-fold higher in the hyperkalemia cohort than in the nonhyperkalemia cohort (95% CI, 1.02 to 1.16; *P* < 0.01; Figure 4). A total of 1682 patients with hyperkalemia and 1550 patients without hyperkalemia died during follow-up. The hazard ratios for additional subanalyses are presented in Supplemental Figure 5.

## Discussion

Multiple factors increase the risk of CKD progression,^35^ but the contribution of hyperkalemia to CKD progression before the development of kidney failure has not been systematically investigated. This observational study addressed this question by comparing CKD progression and mortality among patients with CKD stages 3b/4 with and without hyperkalemia not regularly treated with intestinal K^+^ binders, double-matched on key baseline characteristics including CKD stage (eGFR), and other known risk factors. We found that patients with hyperkalemia were significantly more likely to experience CKD progression and all-cause mortality than those without hyperkalemia. The results were similar in the subset of patients treated with RAASi during baseline, CKD stage at index, and mild hyperkalemia, and when applying alternative definitions of CKD progression.

Data describing hyperkalemia as a factor contributing to CKD progression, rather than as a consequence of advanced CKD, are mixed. In a registry-based study of nondialysis dependent patients with eGFR <60 ml/min per 1.73 m^2^, higher (5.0–5.4 and >5.5 mmol/L) serum K^+^ concentrations were not significantly associated with an increased risk of kidney failure after adjusting for demographic characteristics, comorbidities, and medications.^22^ Conversely, other studies found that hyperkalemia was independently associated with the risk of kidney failure.^24,25^ In the current work, hyperkalemia was associated with increased risks of CKD progression and mortality in patients with CKD stages 3b/4. This is in line with an earlier observation that, in patients with CKD stages 3/4 and hyperkalemia, the adjusted rate of CKD progression to stage 5/kidney failure was higher than in patients without hyperkalemia,^36^ and also confirms the association between hyperkalemia and CKD progression shown in other observational studies.^11,14,19–21,24–26^

The physiologic basis for the link between hyperkalemia and CKD progression may involve excess aldosterone secretion induced by elevated serum K^+^ concentrations.^37,38^ Elevated serum K^+^ concentrations have also been associated with disruptions of myocardial action potentials that can increase the risk of cardiovascular disease, which may further increase the risk of CKD progression.^1,39^ Although additional studies are needed to elucidate the underlying mechanisms of CKD progression related to hyperkalemia, the large magnitude of the estimated effect size, consistent results across various patient subanalyses, and the use of exact and propensity score matching increase our confidence that the observed findings are neither due to chance nor residual confounding.

The association between hyperkalemia and mortality that we observed is also consistent with previous evidence.^4,5,11–16,18,20,21,23^ Hyperkalemia could contribute to mortality risk, not only by promoting CKD progression, but also by decreasing myocardial excitability, increasing the risk of cardiac arrhythmia (specifically, bradycardia, and heart block) and heart failure.^40^ Excess aldosterone is also known to contribute to inflammation and myocardial fibrosis, among other adverse cardiovascular effects.^41^ Hypokalemia (K^+^ <4.0 mmol/L) is also associated with mortality in patients with CKD,^17^ likely through other mechanisms (*e.g*., confounding by high-dose diuretic use in patients with concomitant heart failure or chronic liver disease, ventricular arrhythmia [including torsade de pointes], and/or malnutrition), yielding a U-shaped association between serum K^+^ concentrations and mortality.^4,5,12–14,16,20–22,25,26^ These competing effects may explain the current finding that mortality risk was not increased in patients with mild hyperkalemia relative to those with normokalemia (serum K^+^ ≥3.5 and ≤5.0 mmol/L). The absence of an association between hyperkalemia and all-cause mortality in patients with CKD stage 4 may be attributable to survivorship bias because patients who progressed to stage 4 could not have died while in stage 3b. Alternatively, compensatory mechanisms that have been suggested to enhance tolerance to elevated serum K^+^ concentrations in advanced CKD (*e.g*., altered gastrointestinal secretion of K^+^ or increased insulin-mediated K^+^ reabsorption)^30^ may have negated the harmful effects of hyperkalemia in patients with CKD stage 4. Finally, the smaller sample size of these subanalyses (1707 matched pairs in stage 4 CKD and 2181 matched pairs in mild hyperkalemia) may have also contributed to the observed null associations.

RAASi used to treat CKD can induce hyperkalemia by reducing secretion of, or increasing resistance to, the effects of aldosterone, though RAASi use does not completely inhibit aldosterone activity.^42^ According to Kidney Disease Improving Global Outcomes 2023 guidelines, dose reduction or discontinuation of RAASi should be considered if hyperkalemia persists and remains uncontrolled despite treatment.^43^ However, nonadherence to RAASi treatment has been shown to increase the risk of cardiovascular events and CKD progression and mortality.^7–9,44,45^ In this study, hyperkalemia was associated with adverse CKD outcomes in the subanalysis of patients with baseline RAASi use. The use of intestinal K^+^ binders to manage hyperkalemia can facilitate ongoing use of RAASi therapy,^30,46^ but whether the addition of these agents to a regimen of RAASi with or without sodium-glucose co-transporter-2 inhibitors further attenuates CKD progression is unknown.

This study had multiple strengths. First, we used a large, adjudicated closed claims/electronic medical record database that captured all encounters covered by the insurance provider for a large population of patients with CKD in the United States with or without hyperkalemia. The population was diverse by age, sex, designated race/ethnicity, geography, CKD stage, and the presence or absence of key comorbid conditions, including type 2 diabetes. Second, we used a rigorous study design in which patients in the hyperkalemia cohort were required to have a hyperkalemia diagnosis preceding their diagnosis of CKD to establish temporality, and patients in both cohorts were matched on key clinical characteristics, aiming to minimize potential bias related to reverse causality and to ensure a robust estimate of the association between hyperkalemia and CKD progression. However, as this was an observational study, the risk of residual confounding remains.

There were also several limitations that should be acknowledged. First, the dataset did not contain all variables of interest (*e.g*., cause of death, dietary management, *etc.*). Albuminuria is rarely measured in routine outpatient practice, yet the degree of albuminuria contributes to the risk of CKD progression. Albuminuria is typically assessed *via* the urine albumin-creatinine ratio, which was only available for 27.2% of patients in the final matched sample and, therefore, could not be included as a variable in the exact or propensity score match. As such, we could not determine whether the degree of albuminuria modified the association between hyperkalemia and CKD progression or mortality. Dietary K^+^ intake was also unavailable, and dietary changes in response to elevated serum K^+^ concentrations could contribute to the observed results. Second, eGFR may not be measured on a frequent basis in outpatient practice. eGFR decline was therefore considered as an exploratory end point in this study because it would be better studied in a randomized clinical trial setting with more frequent laboratory monitoring. Third, hyperkalemia was identified using diagnosis codes and serum K^+^ concentrations; as such, mild cases of hyperkalemia may have been undercoded.^47^ Fourth, although the study implemented steps to minimize the potential bias due to reverse causality, the study design makes it difficult to completely disentangle the relation between hyperkalemia and CKD progression, given the potential for a bidirectional association. Finally, the study population was required to have 12 months of continuous enrollment preindex; patients who were enrolled in their health plan for fewer than 12 months (*e.g*., because of changes in employment status) were excluded. The results may not be generalizable to this group if they differed from the overall population of patients with CKD stages 3b/4.

Patients with CKD stages 3b/4 and hyperkalemia experienced significantly higher risks of CKD progression and all-cause mortality than those without hyperkalemia in this observational study. Similar results were observed across subanalyses of patients including those treated with RAASi during baseline. These findings suggest that patients with moderate to advanced (stages 3b/4) CKD may benefit from screening for, and long-term management of, hyperkalemia.

## Supplementary Material

## Data Availability

Data cannot be shared. The datasets generated and analyzed during the current study are not publicly available because they were used pursuant to a data use agreement. The data are available through requests made directly to Optum.

## Supplemental Material

Supplemental Methods

Supplemental References

Supplemental Table 1. Baseline characteristics of the RAASi subanalysis.

Supplemental Table 2. Baseline characteristics of the CKD stage 3 subanalysis.

Supplemental Table 3. Baseline characteristics of the CKD stage 4 subanalysis.

Supplemental Table 4. Baseline characteristics of the mild hyperkalemia subanalysis.

Supplemental Table 5. Baseline characteristics of the recurrent hyperkalemia subanalysis.

Supplemental Table 6. Baseline characteristics of the mild and recurrent hyperkalemia subanalysis.

Supplemental Table 7. Baseline characteristics of the type 2 diabetes subanalysis.

Supplemental Figure 1. Selection of patients with recurrent hyperkalemia (recurrent hyperkalemia subanalysis) and the subset with mild hyperkalemia (mild and recurrent hyperkalemia subanalysis).

Supplemental Figure 2. Selection of patients in the subanalyses.

Supplemental Figure 3. Forest plot of CKD progression hazard ratios in the type 2 diabetes mellitus, recurrent hyperkalemia, and mild and recurrent hyperkalemia subanalyses.

Supplemental Figure 4. Forest plot of secondary and exploratory CKD progression hazard ratios in the overall sample.

Supplemental Figure 5. Forest plot of mortality hazard ratios in the recurrent hyperkalemia and mild and recurrent hyperkalemia subanalyses.
