# Supplementary material for: Hyperkalemia and Risk of CKD Progression: A Propensity Score–Matched Analysis
Source: Kidney360. 2024 Aug 9;5(12):1824–34. doi: 10.34067/KID.0000000000000541 (PMC11687975; doi:10.34067/KID.0000000000000541)
Supplement: Supplementary file 1 [file kidney360-5-1824-s001.pdf]

ASN
American Society of Nephrology
JASN
Journal of the American Society of Nephrology
KIDNEY360
Accessing Our World From Every Angle
CJASN
Clinical Journal of the American Society of Nephrology

ASN Journal Disclosure Form

As per ASN journal policy, I have disclosed any financial relationships or commitments I have held in the past 36 months as included below. I have listed my Current Employer below to indicate there is a relationship requiring disclosure. If no relationship exists, my Current Employer is not listed.

A. Agiro reports the following:
Employer: AstraZeneca; and Ownership Interest: AstraZeneca stocks.

I understand that the information above will be published within the journal article, if accepted, and that failure to comply and/or to accurately and completely report the potential financial conflicts of interest could lead to the following: 1) Prior to publication, article rejection, or 2) Post-publication, sanctions ranging from, but not limited to, issuing a correction, reporting the inaccurate information to the authors' institution, banning authors from submitting work to ASN journals for varying lengths of time, and/or retraction of the published work.

Name: Abiy Agiro
Manuscript ID: K360-2024-000254R1
Manuscript Title: Hyperkalemia and risk of chronic kidney disease progression: A propensity score matched analysis
Date of Completion: July 8, 2024
Disclosure Updated Date: May 1, 2024

ASN American Society of Nephrology | JASN Journal of the American Society of Nephrology | KIDNEY360 Accessing Our World From Every Angle | CJASN Clinical Journal of the American Society of Nephrology

ASN Journal Disclosure Form

As per ASN journal policy, I have disclosed any financial relationships or commitments I have held in the past 36 months as included below. I have listed my Current Employer below to indicate there is a relationship requiring disclosure. If no relationship exists, my Current Employer is not listed.

J. Chen reports the following:
Employer: Analysis Group; and Research Funding: I am an employee at Analysis Group which received research funding for the work under consideration.

I understand that the information above will be published within the journal article, if accepted, and that failure to comply and/or to accurately and completely report the potential financial conflicts of interest could lead to the following: 1) Prior to publication, article rejection, or 2) Post-publication, sanctions ranging from, but not limited to, issuing a correction, reporting the inaccurate information to the authors' institution, banning authors from submitting work to ASN journals for varying lengths of time, and/or retraction of the published work.

Name: Jingyi Chen
Manuscript ID: K360-2024-000254R1
Manuscript Title: Hyperkalemia and risk of chronic kidney disease progression: A propensity score matched analysis
Date of Completion: July 3, 2024
Disclosure Updated Date: May 2, 2024

ASN
American Society of Nephrology
JASN
Journal of the American Society of Nephrology
KIDNEY360
Accessing Our World From Every Angle
CJASN
Clinical Journal of the American Society of Nephrology

ASN Journal Disclosure Form

As per ASN journal policy, I have disclosed any financial relationships or commitments I have held in the past 36 months as included below. I have listed my Current Employer below to indicate there is a relationship requiring disclosure. If no relationship exists, my Current Employer is not listed.

G. Chertow reports the following:
Employer: Stanford University School of Medicine; Consultancy: Akebia, Ardelyx, AstraZeneca, Beren, CalciMedica, Calico, Miromatrix, Panoramic, Sanifit, Toku, Unicycive, Vertex; Ownership Interest: Ardelyx, CloudCath, Durect, DxNow, Eliaz Therapeutics, Outset, Renibus, Unicycive; Research Funding: NIDDK, NIAID, CSL Behring; Advisory or Leadership Role: Board of Directors, Satellite Healthcare, Co-Editor, Brenner & Rector's The Kidney (Elsevier); and Other Interests or Relationships: DSMB service: NIDDK, Aethlon, Bayer, Mineralys, ReCor.

I understand that the information above will be published within the journal article, if accepted, and that failure to comply and/or to accurately and completely report the potential financial conflicts of interest could lead to the following: 1) Prior to publication, article rejection, or 2) Post-publication, sanctions ranging from, but not limited to, issuing a correction, reporting the inaccurate information to the authors' institution, banning authors from submitting work to ASN journals for varying lengths of time, and/or retraction of the published work.

Name: Glenn M Chertow
Manuscript ID: K360-2024-000254R1
Manuscript Title: Hyperkalemia and risk of chronic kidney disease progression: A propensity score matched analysis
Date of Completion: May 28, 2024
Disclosure Updated Date: May 15, 2024

ASN American Society of Nephrology | JASN Journal of the American Society of Nephrology | KIDNEY360 Accessing Our World From Every Angle | CJASN Clinical Journal of the American Society of Nephrology

ASN Journal Disclosure Form

As per ASN journal policy, I have disclosed any financial relationships or commitments I have held in the past 36 months as included below. I have listed my Current Employer below to indicate there is a relationship requiring disclosure. If no relationship exists, my Current Employer is not listed.

E. Colman reports the following:
Employer: AstraZeneca; and Ownership Interest: AstraZeneca.

I understand that the information above will be published within the journal article, if accepted, and that failure to comply and/or to accurately and completely report the potential financial conflicts of interest could lead to the following: 1) Prior to publication, article rejection, or 2) Post-publication, sanctions ranging from, but not limited to, issuing a correction, reporting the inaccurate information to the authors' institution, banning authors from submitting work to ASN journals for varying lengths of time, and/or retraction of the published work.

Name: Ellen Colman
Manuscript ID: K360-2024-000254R1
Manuscript Title: Hyperkalemia and risk of chronic kidney disease progression: A propensity score matched analysis
Date of Completion: July 1, 2024
Disclosure Updated Date: July 1, 2024

ASN American Society of Nephrology
JASN Journal of the American Society of Nephrology
KIDNEY360 Accessing Our World From Every Angle
CJASN Clinical Journal of the American Society of Nephrology

ASN Journal Disclosure Form

As per ASN journal policy, I have disclosed any financial relationships or commitments I have held in the past 36 months as included below. I have listed my Current Employer below to indicate there is a relationship requiring disclosure. If no relationship exists, my Current Employer is not listed.

E. Cook reports the following:
Employer: I am an employee of Analysis Group, Inc. which received consulting fees from AstraZeneca for the study.; Consultancy: I am an employee of Analysis Group, Inc. which received consulting fees from AstraZeneca for the study.; and Research Funding: I am an employee of Analysis Group, Inc. which received consulting fees from AstraZeneca for the study.

I understand that the information above will be published within the journal article, if accepted, and that failure to comply and/or to accurately and completely report the potential financial conflicts of interest could lead to the following: 1) Prior to publication, article rejection, or 2) Post-publication, sanctions ranging from, but not limited to, issuing a correction, reporting the inaccurate information to the authors' institution, banning authors from submitting work to ASN journals for varying lengths of time, and/or retraction of the published work.

Name: Erin Cook
Manuscript ID: K360-2024-000254R1
Manuscript Title: Hyperkalemia and risk of chronic kidney disease progression: A propensity score matched analysis
Date of Completion: May 28, 2024
Disclosure Updated Date: May 28, 2024

ASN American Society of Nephrology JASN Journal of the American Society of Nephrology KIDNEY360 Accessing Our World From Every Angle CJASN Clinical Journal of the American Society of Nephrology

ASN Journal Disclosure Form

As per ASN journal policy, I have disclosed any financial relationships or commitments I have held in the past 36 months as included below. I have listed my Current Employer below to indicate there is a relationship requiring disclosure. If no relationship exists, my Current Employer is not listed.

P. Desai reports the following:
Employer: AstraZeneca; and Ownership Interest: AstraZeneca.

I understand that the information above will be published within the journal article, if accepted, and that failure to comply and/or to accurately and completely report the potential financial conflicts of interest could lead to the following: 1) Prior to publication, article rejection, or 2) Post-publication, sanctions ranging from, but not limited to, issuing a correction, reporting the inaccurate information to the authors' institution, banning authors from submitting work to ASN journals for varying lengths of time, and/or retraction of the published work.

Name: Pooja N. Desai
Manuscript ID: K360-2024-000254R1
Manuscript Title: Hyperkalemia and risk of chronic kidney disease progression: A propensity score matched analysis
Date of Completion: July 1, 2024
Disclosure Updated Date: July 1, 2024

ASN American Society of Nephrology | JASN Journal of the American Society of Nephrology | KIDNEY360 Accessing Our World From Every Angle | CJASN Clinical Journal of the American Society of Nephrology

ASN Journal Disclosure Form

As per ASN journal policy, I have disclosed any financial relationships or commitments I have held in the past 36 months as included below. I have listed my Current Employer below to indicate there is a relationship requiring disclosure. If no relationship exists, my Current Employer is not listed.

A. Greatsinger reports the following:
Employer: Analysis Group; Consultancy: I am an employee of Analysis Group, Inc., which is a consulting firm that received payment from AstraZeneca for the conduction of the study.; and Research Funding: I am an employee of Analysis Group, Inc., which is a consulting firm that received payment from AstraZeneca for the conduction of the study.

I understand that the information above will be published within the journal article, if accepted, and that failure to comply and/or to accurately and completely report the potential financial conflicts of interest could lead to the following: 1) Prior to publication, article rejection, or 2) Post-publication, sanctions ranging from, but not limited to, issuing a correction, reporting the inaccurate information to the authors' institution, banning authors from submitting work to ASN journals for varying lengths of time, and/or retraction of the published work.

Name: Alexandra Greatsinger
Manuscript ID: K360-2024-000254R1
Manuscript Title: Hyperkalemia and risk of chronic kidney disease progression: A propensity score matched analysis
Date of Completion: May 28, 2024
Disclosure Updated Date: April 30, 2024

ASN American Society of Nephrology JASN Journal of the American Society of Nephrology KIDNEY360 Accessing Our World From Every Angle CJASN Clinical Journal of the American Society of Nephrology

ASN Journal Disclosure Form

As per ASN journal policy, I have disclosed any financial relationships or commitments I have held in the past 36 months as included below. I have listed my Current Employer below to indicate there is a relationship requiring disclosure. If no relationship exists, my Current Employer is not listed.

E. Louden reports the following:
Employer: Analysis Group (Boston, MA); Consultancy: AstraZeneca; and Research Funding: AstraZeneca.

I understand that the information above will be published within the journal article, if accepted, and that failure to comply and/or to accurately and completely report the potential financial conflicts of interest could lead to the following: 1) Prior to publication, article rejection, or 2) Post-publication, sanctions ranging from, but not limited to, issuing a correction, reporting the inaccurate information to the authors' institution, banning authors from submitting work to ASN journals for varying lengths of time, and/or retraction of the published work.

Name: Elaine Maria Louden
Manuscript ID: K360-2024-000254R1
Manuscript Title: Hyperkalemia and risk of chronic kidney disease progression: A propensity score matched analysis
Date of Completion: May 29, 2024
Disclosure Updated Date: May 29, 2024

ASN
American Society of Nephrology
JASN
Journal of the American Society of Nephrology
KIDNEY360
Accessing Our World From Every Angle
CJASN
Clinical Journal of the American Society of Nephrology

ASN Journal Disclosure Form

As per ASN journal policy, I have disclosed any financial relationships or commitments I have held in the past 36 months as included below. I have listed my Current Employer below to indicate there is a relationship requiring disclosure. If no relationship exists, my Current Employer is not listed.

F. Mu reports the following:
Employer: Analysis Group; Consultancy: I am an employee of Analysis Group, which received consulting fees from AstraZeneca for the study.; and Research Funding: I am an employee of Analysis Group, which received consulting fees from AstraZeneca for the study.

I understand that the information above will be published within the journal article, if accepted, and that failure to comply and/or to accurately and completely report the potential financial conflicts of interest could lead to the following: 1) Prior to publication, article rejection, or 2) Post-publication, sanctions ranging from, but not limited to, issuing a correction, reporting the inaccurate information to the authors' institution, banning authors from submitting work to ASN journals for varying lengths of time, and/or retraction of the published work.

Name: Fan Mu
Manuscript ID: K360-2024-000254R1
Manuscript Title: Hyperkalemia and risk of chronic kidney disease progression: A propensity score matched analysis
Date of Completion: May 28, 2024
Disclosure Updated Date: May 28, 2024

ASN American Society of Nephrology · JASN Journal of the American Society of Nephrology · KIDNEY360 Accessing Our World From Every Angle · CJASN Clinical Journal of the American Society of Nephrology

ASN Journal Disclosure Form

As per ASN journal policy, I have disclosed any financial relationships or commitments I have held in the past 36 months as included below. I have listed my Current Employer below to indicate there is a relationship requiring disclosure. If no relationship exists, my Current Employer is not listed.

A. Zhao reports the following:
Employer: Analysis Group; and Consultancy: I am an employee of Analysis Group, Inc., which is a consulting firm that received payment from AstraZeneca for the conduction of the study.

I understand that the information above will be published within the journal article, if accepted, and that failure to comply and/or to accurately and completely report the potential financial conflicts of interest could lead to the following: 1) Prior to publication, article rejection, or 2) Post-publication, sanctions ranging from, but not limited to, issuing a correction, reporting the inaccurate information to the authors' institution, banning authors from submitting work to ASN journals for varying lengths of time, and/or retraction of the published work.

Name: Angela Zhao
Manuscript ID: K360-2024-000254R1
Manuscript Title: Hyperkalemia and risk of chronic kidney disease progression: A propensity score matched analysis
Date of Completion: July 1, 2024
Disclosure Updated Date: July 1, 2024
