# Supplementary material for: Hyperkalemia and Risk of CKD Progression: A Propensity Score–Matched Analysis
Source: Kidney360. 2024 Aug 9;5(12):1824–34. doi: 10.34067/KID.0000000000000541 (PMC11687975; doi:10.34067/KID.0000000000000541)
Supplement: Supplementary file 2 [file kidney360-5-1824-s002.pdf]

SUPPLEMENTAL MATERIALS

Table of Contents

1. **Supplemental Methods**
2. **Supplemental References**
3. **Supplemental Table S1**. Baseline characteristics of the RAASi sub-analysis
4. **Supplemental Table S2.** Baseline characteristics of the CKD stage 3 sub-analysis
5. **Supplemental Table S3.** Baseline characteristics of the CKD stage 4 sub-analysis
6. **Supplemental Table S4.** Baseline characteristics of the mild hyperkalemia sub-analysis
7. **Supplemental Table S5.** Baseline characteristics of the recurrent hyperkalemia sub-analysis
8. **Supplemental Table S6.** Baseline characteristics of the mild and recurrent hyperkalemia sub-analysis
9. **Supplemental Table S7.** Baseline characteristics of the type 2 diabetes sub-analysis
10. **Supplemental Figure S1.** Selection of patients with recurrent hyperkalemia (recurrent hyperkalemia sub-analysis) and the subset with mild hyperkalemia (mild and recurrent hyperkalemia sub-analysis).
11. **Supplemental Figure S2.** Selection of patients in the sub-analyses
12. **Supplemental Figure S3.** Forest plot of CKD progression hazard ratios in the type 2 diabetes mellitus, recurrent hyperkalemia, and mild and recurrent hyperkalemia sub-analyses.
13. **Supplemental Figure S4.** Forest plot of secondary and exploratory CKD progression hazard ratios in the overall sample.
14. **Supplemental Figure S5.** Forest plot of mortality hazard ratios in the recurrent hyperkalemia and mild and recurrent hyperkalemia sub-analyses.

Supplemental methods

Patient sub-analyses

Analyses were performed in the overall population and in the following patient sub-analyses: patients with RAASi use during baseline; CKD stage 3 at index (based on diagnosis codes); CKD stage 4 at index (based on diagnosis codes); and mild hyperkalemia (i.e., serum K+ value closest to the index date during baseline of >5.0 to ≤5.5 mmol/L) (Bakris 2015; Israni 2021; National Kidney Foundation 2023; Weir 2016). Additional sub-analysis analyses were performed in patients with type 2 diabetes mellitus during baseline; recurrent hyperkalemia; and mild and recurrent hyperkalemia.

Recurrent hyperkalemia was defined as having ≥2 hyperkalemia events occurring >7 days but ≤1 year apart, and ≥1 diagnosis code of hyperkalemia before the index date. A hyperkalemia event was defined as either a serum K+ concentration >5.0 mmol/L or hyperkalemia diagnosis in any setting; laboratory values and claims ≤7 days apart were considered as a single event. The index date for patients with recurrent hyperkalemia was the first CKD stage 3/4 diagnosis with ≥2 hyperkalemia events in the preceding 12 months. Non-hyperkalemia patients for the recurrent hyperkalemia analysis were the same as those in the overall analysis. Patients meeting both mild and recurrent hyperkalemia definitions constituted the mild and recurrent hyperkalemia sub-analysis.

REFERENCES

Bakris GL, Pitt B, Weir MR, et al. Effect of patiromer on serum potassium level in patients with hyperkalemia and diabetic kidney disease: The AMETHYST-DN randomized clinical trial. JAMA. 2015;314(2):151–161.

Israni R, Betts KA, Mu F, Davis J, Wang J, Anzalone D, Uwaifo GI, Szerlip H, Fonseca V, Wu E. Determinants of Hyperkalemia Progression Among Patients with Mild Hyperkalemia. Adv Ther. 2021 Nov;38(11):5596-5608.

National Kidney Foundation. Stages of Chronic Kidney Disease. Available at: https://www.kidney.org/atoz/content/stages-chronic-kidney-disease-ckd. Accessed 21 September 2023.

Weir MR. Current and future treatment options for managing hyperkalemia. Kidney Int Suppl (2011). 2016;6(1):29–34.

Supplemental Table S1. Baseline characteristics of the RAASi sub-analysis

	Cohort		
	**Hyperkalemia (N=5,022)**	**Non-Hyperkalemia (N=5,022)**	**SMD[1]**
**Demographics**			
Age, years	73.9±11.4	73.9±11.4	0.002
Female	2,720 (54.2)	2,660 (53.0)	0.024
Race			0.037
African American	748 (14.9)	806 (16.0)	
Asian	85 (1.7)	82 (1.6)	
Caucasian	3,815 (76.0)	3,742 (74.5)	
Other/unknown	374 (7.4)	392 (7.8)	
Medical insurance type			0.073
Commercial	931 (18.5)	951 (18.9)	
Medicaid	321 (6.4)	365 (7.3)	
Medicare	3,538 (70.5)	3,538 (70.5)	
Unknown	232 (4.6)	168 (3.3)	
**Clinical characteristics**			
BMI, kg/m^2	31.4±7.7	32.0±7.6	0.076
CKD stage			
Diagnosis code at index			0.000
Stage 3	3,831 (76.3%)	3,831 (76.3%)	
Stage 4	1,191 (23.7%)	1,191 (23.7%)	
eGFR closest to index[2], ml/min/1.73 m^2			0.000
Stage 3b (30 to <45)	3,633 (72.3)	3,633 (72.3)	
Stage 4 (15 to <30)	1,389 (27.7)	1,389 (27.7)	
Mean ± SD	34.2 ± 7.3	34.3 ± 7.4	0.006
Median (25%, 75% range25%, 75% range)	35.4 (29.2, 40.2)	35.5 (29.2, 40.4)	
Hyperkalemia			
Total number of hyperkalemia events during baseline			
Mean ± SD	3.4 ± 1.9	0.0 ± 0.0	2.579
Median (25%, 75% range)	3.0 (2.0, 4.0)	0.0 (0.0, 0.0)	
Serum K+ lab concentration closest to index date, mmol/L			
Mean ± SD	5.0 ± 0.6	4.2 ± 0.4	1.618
Median (25%, 75% range)	5.1 (4.7, 5.4)	4.2 (3.9, 4.5)	
Hyperkalemia severity			1.575
≤5	2,242 (44.6)	5,022 (100.0)	
>5 to <5.5	1,656 (33.0)	0 (0.0)	
5.5 to <6	906 (18.0)	0 (0.0)	
≥6	218 (4.3)	0 (0.0)	
Most severe serum K+ concentration during baseline, mmol/L			
Mean ± SD	5.6 ± 0.4	4.5 ± 0.3	3.034
Median (25%, 75% range)	5.5 (5.3, 5.8)	4.5 (4.2, 4.7)	
Hyperkalemia severity			0.817
≤5	0 (0.0)	5,022 (100.0)	
>5 to <5.5	2,229 (44.4)	0 (0.0)	
5.5 to <6	2,112 (42.1)	0 (0.0)	
≥6	681 (13.6)	0 (0.0)	
Comorbidities			
Charlson comorbidity index	3.6±2.2	3.6±2.1	0.005

Hyperkalemia-related comorbidities			
Acute kidney injury	1,966 (39.1)	1,983 (39.5)	0.007
Coronary artery disease	2,031 (40.4)	2,072 (41.3)	0.017
Type 2 diabetes	3,207 (63.9)	3,226 (64.2)	0.008
Hypertension	4,754 (94.7)	4,747 (94.5)	0.006
Edema	1,626 (32.4)	1,586 (31.6)	0.017
Cardiovascular-related comorbidities			
Cerebrovascular disease	1,052 (20.9)	1,075 (21.4)	0.011
Congestive heart failure	1,940 (38.6)	1,911 (38.1)	0.012
Myocardial infarction	702 (14.0)	693 (13.8)	0.005
Peripheral vascular disease	1,717 (34.2)	1,574 (31.3)	0.061
**Medication use**			
Any RAASi	5,022 (100.0)	5,022 (100.0)	0.000
ACE inhibitor	2,978 (59.3)	2,505 (49.9)	0.190
ARB	2,017 (40.2)	2,464 (49.1)	0.180
ARN inhibitor	124 (2.5)	93 (1.9)	0.042
MRA	1,098 (21.9)	698 (13.9)	0.209
Beta blockers	3,264 (65.0)	3,297 (65.7)	0.014
**Healthcare resource utilization**			
Any inpatient visits	1,651 (32.9)	1,570 (31.3)	0.035
Any CKD-related inpatient visit	1,400 (27.9)	1,267 (25.2)	0.060
Any emergency department visits	1,461 (29.1)	1,416 (28.2)	0.020

Values are reported as mean±SD, median (25%, 75% range), or n (%).

Abbreviations: ACE, angiotensin-converting enzyme; ARB, angiotensin receptor blocker; ARN, angiotensin receptor/neprilysin; BMI, body mass index; CKD, chronic kidney disease; eGFR, estimated glomerular filtration rate; MRA, mineralocorticoid receptor antagonist; RAASi, renin-angiotensin-aldosterone system inhibitor; SD, standard deviation; SMD, standardized mean difference.

Notes:

[1] SMD values >0.2 indicate an imbalance between cohorts.

[2] eGFR was assessed in the outpatient setting.

Supplemental Table S2. Baseline characteristics of the CKD stage 3 sub-analysis

	Cohort		
	**Hyperkalemia (N=4,912)**	**Non-Hyperkalemia (N=4,912)**	**SMD[1]**
**Demographics**			
Age, years	74.9±10.8	74.9±11.0	0.001
Female	2,639 (53.7)	2,593 (52.8)	0.019
Race			0.012
African American	715 (14.6)	698 (14.2)	
Asian	69 (1.4)	70 (1.4)	
Caucasian	3,784 (77.0)	3,808 (77.5)	
Other/unknown	344 (7.0)	336 (6.8)	
Medical insurance type			0.034
Commercial	875 (17.8)	890 (18.1)	
Medicaid	276 (5.6)	291 (5.9)	
Medicare	3,553 (72.3)	3,553 (72.3)	
Unknown	208 (4.2)	178 (3.6)	
**Clinical characteristics**			
BMI, kg/m^2	31.0±7.7	31.7±7.6	0.088
CKD stage			
Diagnosis code at index			0.000
Stage 3	4,912 (100.0%)	4,912 (100.0%)	
Stage 4	0 (0.0%)	0 (0.0%)	
eGFR closest to index[2], ml/min/1.73 m^2			0.000
Stage 3b (30 to <45)	4,263 (86.8)	4,263 (86.8)	
Stage 4 (15 to <30)	649 (13.2)	649 (13.2)	
Mean ± SD	36.6 ± 5.8	36.8 ± 5.8	0.019
Median (25%, 75% range)	37.5 (33.2, 41.3)	37.7 (33.2, 41.5)	
Hyperkalemia			
Total number of hyperkalemia events during baseline			
Mean ± SD	3.4 ± 2.0	0.0 ± 0.0	2.446
Median (25%, 75% range)	3.0 (2.0, 4.0)	0.0 (0.0, 0.0)	
Serum K+ lab concentration closest to index date, mmol/L			
Mean ± SD	5.0 ± 0.6	4.2 ± 0.4	1.563
Median (25%, 75% range)	5.1 (4.6, 5.4)	4.2 (3.9, 4.5)	
Hyperkalemia severity			1.490
≤5	2,328 (47.4)	4,912 (100.0)	
>5 to <5.5	1,556 (31.7)	0 (0.0)	
5.5 to <6	839 (17.1)	0 (0.0)	
≥6	189 (3.8)	0 (0.0)	
Most severe serum K+ concentration during baseline, mmol/L			
Mean ± SD	5.6 ± 0.4	4.4 ± 0.3	3.062
Median (25%, 75% range)	5.5 (5.3, 5.8)	4.5 (4.2, 4.7)	
Hyperkalemia severity			0.876
≤5	0 (0.0)	4,912 (100.0)	
>5 to <5.5	2,237 (45.5)	0 (0.0)	
5.5 to <6	2,050 (41.7)	0 (0.0)	
≥6	625 (12.7)	0 (0.0)	
Comorbidities			
Charlson comorbidity index	3.6±2.2	3.6±2.2	0.014

Hyperkalemia-related comorbidities			
Acute kidney injury	1,974 (40.2)	1,933 (39.4)	0.017
Coronary artery disease	2,033 (41.4)	2,011 (40.9)	0.009
Type 2 diabetes	2,974 (60.5)	2,977 (60.6)	0.001
Hypertension	4,550 (92.6)	4,524 (92.1)	0.020
Edema	1,562 (31.8)	1,528 (31.1)	0.015
Cardiovascular-related comorbidities			
Cerebrovascular disease	1,044 (21.3)	1,102 (22.4)	0.029
Congestive heart failure	1,889 (38.5)	1,818 (37.0)	0.030
Myocardial infarction	725 (14.8)	679 (13.8)	0.027
Peripheral vascular disease	1,757 (35.8)	1,574 (32.0)	0.079
**Medication use**			
Any RAASi	3,831 (78.0)	3,831 (78.0)	0.000
ACE inhibitor	2,316 (47.1)	1,925 (39.2)	0.161
ARB	1,507 (30.7)	1,860 (37.9)	0.152
ARN inhibitor	99 (2.0)	67 (1.4)	0.051
MRA	883 (18.0)	548 (11.2)	0.194
Beta blockers	3,003 (61.1)	3,022 (61.5)	0.008
**Healthcare resource utilization**			
Any inpatient visits	1,747 (35.6)	1,717 (35.0)	0.013
Any CKD-related inpatient visit	1,469 (29.9)	1,376 (28.0)	0.042
Any emergency department visits	1,396 (28.4)	1,384 (28.2)	0.005

Values are reported as mean±SD median (25%, 75% range), or n (%).

Abbreviations: ACE, angiotensin-converting enzyme; ARB, angiotensin receptor blocker; ARN, angiotensin receptor/neprilysin; BMI, body mass index; CKD, chronic kidney disease; eGFR, estimated glomerular filtration rate; MRA, mineralocorticoid receptor antagonist; RAASi, renin-angiotensin-aldosterone system inhibitor; SD, standard deviation; SMD, standardized mean difference.

Notes:

[1] SMD values >0.2 indicate an imbalance between cohorts.

[2] eGFR was assessed in the outpatient setting.

Supplemental Table S3. Baseline characteristics of the CKD stage 4 sub-analysis

	Cohort		
	**Hyperkalemia (N=1,707)**	**Non-Hyperkalemia (N=1,707)**	**SMD[1]**
**Demographics**			
Age, years	73.5±12.4	73.5±12.4	0.000
Female	869 (50.9)	915 (53.6)	0.054
Race			0.060
African American	257 (15.1)	294 (17.2)	
Asian	37 (2.2)	33 (1.9)	
Caucasian	1,244 (72.9)	1,215 (71.2)	
Other/unknown	169 (9.9)	165 (9.7)	
Medical insurance type			0.116
Commercial	365 (21.4)	350 (20.5)	
Medicaid	110 (6.4)	151 (8.8)	
Medicare	1,146 (67.1)	1,146 (67.1)	
Unknown	86 (5.0)	60 (3.5)	
**Clinical characteristics**			
BMI, kg/m^2	30.3±7.2	30.9±7.5	0.079
CKD stage			
Diagnosis code at index			0.000
Stage 3	0 (0.0%)	0 (0.0%)	
Stage 4	1,707 (100.0%)	1,707 (100.0%)	
eGFR closest to index[2], ml/min/1.73 m^2			0.000
Stage 3b (30 to <45)	452 (26.5)	452 (26.5)	
Stage 4 (15 to <30)	1,255 (73.5)	1,255 (73.5)	
Mean ± SD	26.5 ± 6.4	26.5 ± 6.4	0.014
Median (25%, 75% range)	26.0 (21.7, 30.4)	25.8 (21.7, 30.4)	
Hyperkalemia			
Total number of hyperkalemia events during baseline			
Mean ± SD	3.4 ± 2.1	0.0 ± 0.0	2.296
Median (25%, 75% range)	3.0 (2.0, 4.0)	0.0 (0.0, 0.0)	
Serum K+ lab concentration closest to index date, mmol/L			
Mean ± SD	5.1 ± 0.6	4.2 ± 0.4	1.827
Median (25%, 75% range)	5.2 (4.8, 5.4)	4.2 (3.9, 4.5)	
Hyperkalemia severity			1.775
≤5	663 (38.8)	1,707 (100.0)	
>5 to <5.5	625 (36.6)	0 (0.0)	
5.5 to <6	341 (20.0)	0 (0.0)	
≥6	78 (4.6)	0 (0.0)	
Most severe serum K+ concentration during baseline, mmol/L			
Mean ± SD	5.6 ± 0.4	4.4 ± 0.4	3.099
Median (25%, 75% range)	5.5 (5.3, 5.8)	4.5 (4.2, 4.7)	
Hyperkalemia severity			0.864
≤5	0 (0.0)	1,707 (100.0)	
>5 to <5.5	772 (45.2)	0 (0.0)	
5.5 to <6	715 (41.9)	0 (0.0)	
≥6	220 (12.9)	0 (0.0)	
Comorbidities			
Charlson comorbidity index	3.5±2.1	3.5±2.1	0.025

Hyperkalemia-related comorbidities			
Acute kidney injury	653 (38.3)	668 (39.1)	0.018
Coronary artery disease	619 (36.3)	676 (39.6)	0.069
Type 2 diabetes	1,001 (58.6)	1,020 (59.8)	0.023
Hypertension	1,576 (92.3)	1,579 (92.5)	0.007
Edema	522 (30.6)	541 (31.7)	0.024
Cardiovascular-related comorbidities			
Cerebrovascular disease	317 (18.6)	332 (19.4)	0.022
Congestive heart failure	598 (35.0)	660 (38.7)	0.075
Myocardial infarction	187 (11.0)	232 (13.6)	0.080
Peripheral vascular disease	525 (30.8)	515 (30.2)	0.013
**Medication use**			
Any RAASi	1,191 (69.8)	1,191 (69.8)	0.000
ACE inhibitor	662 (38.8)	580 (34.0)	0.100
ARB	510 (29.9)	604 (35.4)	0.118
ARN inhibitor	25 (1.5)	26 (1.5)	0.005
MRA	215 (12.6)	150 (8.8)	0.123
Beta blockers	1,033 (60.5)	1,080 (63.3)	0.057
**Healthcare resource utilization**			
Any inpatient visits	440 (25.8)	371 (21.7)	0.095
Any CKD-related inpatient visit	393 (23.0)	327 (19.2)	0.095
Any emergency department visits	512 (30.0)	493 (28.9)	0.024

Values are reported as mean±SD median (25%, 75% range), or n (%).

Abbreviations: ACE, angiotensin-converting enzyme; ARB, angiotensin receptor blocker; ARN, angiotensin receptor/neprilysin; BMI, body mass index; CKD, chronic kidney disease; eGFR, estimated glomerular filtration rate; MRA, mineralocorticoid receptor antagonist; RAASi, renin-angiotensin-aldosterone system inhibitor; SD, standard deviation; SMD, standardized mean difference.

Notes:

[1] SMD values >0.2 indicate an imbalance between cohorts.

[2] eGFR was assessed in the outpatient setting.

Supplemental Table S4. Baseline characteristics of the mild hyperkalemia sub-analysis

	Cohort		
	**Hyperkalemia (N=2,181)**	**Non-Hyperkalemia (N=2,181)**	**SMD[1]**
**Demographics**			
Age, years	74.5±11.4	74.3±11.6	0.017
Female	1,124 (51.5)	1,138 (52.2)	0.013
Race			0.050
African American	292 (13.4)	330 (15.1)	
Asian	34 (1.6)	35 (1.6)	
Caucasian	1,677 (76.9)	1,639 (75.1)	
Other/unknown	178 (8.2)	177 (8.1)	
Medical insurance type			0.059
Commercial	411 (18.8)	429 (19.7)	
Medicaid	135 (6.2)	140 (6.4)	
Medicare	1,544 (70.8)	1,544 (70.8)	
Unknown	91 (4.2)	68 (3.1)	
**Clinical characteristics**			
BMI, kg/m^2	31.0±7.4	31.5±7.8	0.062
CKD stage			
Diagnosis code at index			0.000
Stage 3	1,556 (71.3%)	1,556 (71.3%)	
Stage 4	625 (28.7%)	625 (28.7%)	
eGFR closest to index[2], ml/min/1.73 m^2			0.000
Stage 3b (30 to <45)	1,478 (67.8)	1,478 (67.8)	
Stage 4 (15 to <30)	703 (32.2)	703 (32.2)	
Mean ± SD	33.6 ± 7.4	33.6 ± 7.8	0.002
Median (25%, 75% range)	34.5 (28.2, 39.8)	34.8 (27.7, 40.2)	
Hyperkalemia			
Total number of hyperkalemia events during baseline			
Mean ± SD	3.5 ± 2.2	0.0 ± 0.0	2.237
Median (25%, 75% range)	3.0 (2.0, 4.0)	0.0 (0.0, 0.0)	
Serum K+ lab concentration closest to index date, mmol/L			
Mean ± SD	5.2 ± 0.1	4.2 ± 0.4	3.431
Median (25%, 75% range)	5.2 (5.1, 5.3)	4.2 (4.0, 4.5)	
Hyperkalemia severity			N/A
≤5	0 (0.0)	2,181 (100.0)	
>5 to <5.5	2,181 (100.0)	0 (0.0)	
5.5 to <6	0 (0.0)	0 (0.0)	
≥6	0 (0.0)	0 (0.0)	
Most severe serum K+ concentration during baseline, mmol/L			
Mean ± SD	5.4 ± 0.3	4.4 ± 0.3	3.023
Median (25%, 75% range)	5.4 (5.2, 5.6)	4.5 (4.2, 4.7)	
Hyperkalemia severity			1.517
≤5	0 (0.0)	2,181 (100.0)	
>5 to <5.5	1,463 (67.1)	0 (0.0)	
5.5 to <6	562 (25.8)	0 (0.0)	
≥6	156 (7.2)	0 (0.0)	
Comorbidities			
Charlson comorbidity index	3.4±2.1	3.5±2.1	0.036
Hyperkalemia-related comorbidities			

Acute kidney injury	785 (36.0)	837 (38.4)	0.049
Coronary artery disease	823 (37.7)	841 (38.6)	0.017
Type 2 diabetes	1,337 (61.3)	1,319 (60.5)	0.017
Hypertension	2,004 (91.9)	1,998 (91.6)	0.010
Edema	601 (27.6)	658 (30.2)	0.058
Cardiovascular-related comorbidities			
Cerebrovascular disease	431 (19.8)	463 (21.2)	0.036
Congestive heart failure	734 (33.7)	765 (35.1)	0.030
Myocardial infarction	249 (11.4)	260 (11.9)	0.016
Peripheral vascular disease	686 (31.5)	642 (29.4)	0.044
**Medication use**			
Any RAASi	1,656 (75.9)	1,656 (75.9)	0.000
ACE inhibitor	962 (44.1)	824 (37.8)	0.129
ARB	689 (31.6)	815 (37.4)	0.122
ARN inhibitor	38 (1.7)	24 (1.1)	0.054
MRA	309 (14.2)	235 (10.8)	0.103
Beta blockers	1,278 (58.6)	1,308 (60.0)	0.028
**Healthcare resource utilization**			
Any inpatient visits	615 (28.2)	604 (27.7)	0.011
Any CKD-related inpatient visit	520 (23.8)	490 (22.5)	0.033
Any emergency department visits	556 (25.5)	618 (28.3)	0.064

Values are reported as mean±SD median (25%, 75% range), or n (%).

Abbreviations: ACE, angiotensin-converting enzyme; ARB, angiotensin receptor blocker; ARN, angiotensin receptor/neprilysin; BMI, body mass index; CKD, chronic kidney disease; eGFR, estimated glomerular filtration rate; MRA, mineralocorticoid receptor antagonist; RAASi, renin-angiotensin-aldosterone system inhibitor; SD, standard deviation; SMD, standardized mean difference.

Notes:

[1] SMD values >0.2 indicate an imbalance between cohorts.

[2] eGFR was assessed in the outpatient setting.

Supplemental Table S5. Baseline characteristics of the recurrent hyperkalemia sub-analysis

	Cohort		
	**Hyperkalemia (N=6,922)**	**Non-Hyperkalemia (N=6,922)**	**SMD[1]**
**Demographics**			
Age, years	74.4±11.2	74.3±11.5	0.007
Female	3,594 (51.9)	3,642 (52.6)	0.014
Race			0.020
African American	1,017 (14.7)	986 (14.2)	
Asian	113 (1.6)	117 (1.7)	
Caucasian	5,239 (75.7)	5,234 (75.6)	
Other/unknown	553 (8.0)	585 (8.5)	
Medical insurance type			0.021
Commercial	1,285 (18.6)	1,298 (18.8)	
Medicaid	405 (5.9)	419 (6.1)	
Medicare	4,935 (71.3)	4,935 (71.3)	
Unknown	297 (4.3)	270 (3.9)	
**Clinical characteristics**			
BMI, kg/m^2	30.9±7.7	31.4±7.3	0.069
CKD stage			
Diagnosis code at index			0.000
Stage 3	5,071 (73.3%)	5,071 (73.3%)	
Stage 4	1,851 (26.7%)	1,851 (26.7%)	
eGFR closest to index[2], ml/min/1.73 m^2			0.000
Stage 3b (30 to <45)	4,879 (70.5)	4,879 (70.5)	
Stage 4 (15 to <30)	2,043 (29.5)	2,043 (29.5)	
Mean ± SD	34.0 ± 7.4	34.0 ± 7.6	0.003
Median (25%, 75% range)	35.1 (28.6, 40.2)	35.2 (28.5, 40.3)	
Hyperkalemia			
Total number of hyperkalemia events during baseline			
Mean ± SD	3.7 ± 2.4	0.0 ± 0.0	2.171
Median (25%, 75% range)	3.0 (2.0, 4.0)	0.0 (0.0, 0.0)	
Serum K+ lab concentration closest to index date, mmol/L			
Mean ± SD	5.1 ± 0.6	4.2 ± 0.4	1.729
Median (25%, 75% range)	5.1 (4.7, 5.4)	4.2 (3.9, 4.5)	
Hyperkalemia severity			1.659
≤5	2,913 (42.1)	6,922 (100.0)	
>5 to <5.5	2,551 (36.9)	0 (0.0)	
5.5 to <6	1,171 (16.9)	0 (0.0)	
≥6	287 (4.1)	0 (0.0)	
Most severe serum K+ concentration during baseline, mmol/L			
Mean ± SD	5.6 ± 0.4	4.4 ± 0.4	3.027
Median (25%, 75% range)	5.5 (5.3, 5.8)	4.5 (4.2, 4.7)	
Hyperkalemia severity			0.850
≤5	0 (0.0)	6,922 (100.0)	
>5 to <5.5	3,166 (45.7)	0 (0.0)	
5.5 to <6	2,849 (41.2)	0 (0.0)	
≥6	907 (13.1)	0 (0.0)	
Comorbidities			
Charlson comorbidity index	3.7±2.2	3.6±2.2	0.025
Hyperkalemia-related comorbidities			

Acute kidney injury	2,786 (40.2)	2,779 (40.1)	0.002
Coronary artery disease	2,865 (41.4)	2,805 (40.5)	0.018
Type 2 diabetes	4,219 (61.0)	4,248 (61.4)	0.009
Hypertension	6,424 (92.8)	6,358 (91.9)	0.036
Edema	2,246 (32.4)	2,239 (32.3)	0.002
Cardiovascular-related comorbidities			
Cerebrovascular disease	1,513 (21.9)	1,513 (21.9)	0.000
Congestive heart failure	2,666 (38.5)	2,690 (38.9)	0.007
Myocardial infarction	987 (14.3)	935 (13.5)	0.022
Peripheral vascular disease	2,461 (35.6)	2,239 (32.3)	0.068
**Medication use**			
Any RAASi	5,219 (75.4)	5,219 (75.4)	0.000
ACE inhibitor	3,141 (45.4)	2,569 (37.1)	0.168
ARB	2,056 (29.7)	2,595 (37.5)	0.165
ARN inhibitor	130 (1.9)	112 (1.6)	0.020
MRA	1,147 (16.6)	710 (10.3)	0.186
Beta blockers	4,248 (61.4)	4,230 (61.1)	0.005
**Healthcare resource utilization**			
Any inpatient visits	2,332 (33.7)	2,187 (31.6)	0.045
Any CKD-related inpatient visit	1,971 (28.5)	1,770 (25.6)	0.065
Any emergency department visits	1,979 (28.6)	1,919 (27.7)	0.019

Values are reported as mean±SD median (25%, 75% range), or n (%).

Abbreviations: ACE, angiotensin-converting enzyme; ARB, angiotensin receptor blocker; ARN, angiotensin receptor/neprilysin; BMI, body mass index; CKD, chronic kidney disease; eGFR, estimated glomerular filtration rate; MRA, mineralocorticoid receptor antagonist; RAASi, renin-angiotensin-aldosterone system inhibitor; SD, standard deviation; SMD, standardized mean difference.

Notes:

[1] SMD values >0.2 indicate an imbalance between cohorts.

[2] eGFR was assessed in the outpatient setting.

Supplemental Table S6. Baseline characteristics of the mild and recurrent hyperkalemia sub-analysis

	Cohort		
	Hyperkalemia (N=2,551)	Non-Hyperkalemia (N=2,551)	SMD[1]
**Demographics**			
Age, years	74.2±11.6	74.4±11.5	0.022
Female	1,276 (50.0)	1,370 (53.7)	0.074
Race			0.019
African American	366 (14.3)	358 (14.0)	
Asian	39 (1.5)	44 (1.7)	
Caucasian	1,921 (75.3)	1,919 (75.2)	
Other/unknown	225 (8.8)	230 (9.0)	
Medical insurance type			0.040
Commercial	485 (19.0)	471 (18.5)	
Medicaid	144 (5.6)	166 (6.5)	
Medicare	1,811 (71.0)	1,811 (71.0)	
Unknown	111 (4.4)	103 (4.0)	
**Clinical characteristics**			
BMI, kg/m^2	31.0±7.5	31.4±7.5	0.050
CKD stage			
Diagnosis code at index			0.000
Stage 3	1,831 (71.8%)	1,831 (71.8%)	
Stage 4	720 (28.2%)	720 (28.2%)	
eGFR closest to index[2], ml/min/1.73 m^2			0.000
Stage 3b (30 to <45)	1,721 (67.5)	1,721 (67.5)	
Stage 4 (15 to <30)	830 (32.5)	830 (32.5)	
Mean ± SD	33.6 ± 7.6	33.5 ± 7.7	0.010
Median (25%, 75% range)	34.5 (28.0, 40.0)	34.8 (27.7, 40.0)	
Hyperkalemia			
Total number of hyperkalemia events during baseline			
Mean ± SD	3.7 ± 2.4	0.0 ± 0.0	2.224
Median (25%, 75% range)	3.0 (2.0, 4.0)	0.0 (0.0, 0.0)	
Serum K+ lab concentration closest to index date, mmol/L			
Mean ± SD	5.2 ± 0.1	4.2 ± 0.4	3.397
Median (25%, 75% range)	5.2 (5.1, 5.3)	4.2 (3.9, 4.5)	
Hyperkalemia severity			N/A
≤5	0 (0.0)	2,551 (100.0)	
>5 to <5.5	2,551 (100.0)	0 (0.0)	
5.5 to <6	0 (0.0)	0 (0.0)	
≥6	0 (0.0)	0 (0.0)	
Most severe serum K+ concentration during baseline, mmol/L			
Mean ± SD	5.4 ± 0.3	4.4 ± 0.4	3.002
Median (25%, 75% range)	5.4 (5.2, 5.6)	4.5 (4.2, 4.7)	
Hyperkalemia severity			1.561
≤5	0 (0.0)	2,551 (100.0)	
>5 to <5.5	1,671 (65.5)	0 (0.0)	
5.5 to <6	708 (27.8)	0 (0.0)	
≥6	172 (6.7)	0 (0.0)	
Comorbidities			

Charlson comorbidity index	3.5±2.1	3.6±2.1	0.015
Hyperkalemia-related comorbidities			
Acute kidney injury	970 (38.0)	1,045 (41.0)	0.060
Coronary artery disease	1,032 (40.5)	1,028 (40.3)	0.003
Type 2 diabetes	1,599 (62.7)	1,573 (61.7)	0.021
Hypertension	2,357 (92.4)	2,333 (91.5)	0.035
Edema	774 (30.3)	803 (31.5)	0.025
Cardiovascular-related comorbidities			
Cerebrovascular disease	547 (21.4)	551 (21.6)	0.004
Congestive heart failure	932 (36.5)	959 (37.6)	0.022
Myocardial infarction	314 (12.3)	339 (13.3)	0.029
Peripheral vascular disease	886 (34.7)	813 (31.9)	0.061
**Medication use**			
Any RAASi	1,915 (75.1)	1,915 (75.1)	0.000
ACE inhibitor	1,146 (44.9)	950 (37.2)	0.157
ARB	771 (30.2)	936 (36.7)	0.137
ARN inhibitor	44 (1.7)	31 (1.2)	0.042
MRA	379 (14.9)	254 (10.0)	0.149
Beta blockers	1,536 (60.2)	1,520 (59.6)	0.013
**Healthcare resource utilization**			
Any inpatient visits	795 (31.2)	744 (29.2)	0.044
Any CKD-related inpatient visit	681 (26.7)	584 (22.9)	0.088
Any emergency department visits	662 (26.0)	699 (27.4)	0.033

Values are reported as mean±SD median (25%, 75% range), or n (%).

Abbreviations: ACE, angiotensin-converting enzyme; ARB, angiotensin receptor blocker; ARN, angiotensin receptor/neprilysin; BMI, body mass index; CKD, chronic kidney disease; eGFR, estimated glomerular filtration rate; MRA, mineralocorticoid receptor antagonist; RAASi, renin-angiotensin-aldosterone system inhibitor; SD, standard deviation; SMD, standardized mean difference.

Notes:

[1] SMD values >0.2 indicate an imbalance between cohorts.

[2] eGFR was assessed in the outpatient setting.

Supplemental Table S7. Baseline characteristics of the type 2 diabetes sub-analysis

	Cohort		
	**Hyperkalemia (N=2,718)**	**Non-Hyperkalemia (N=2,718)**	**SMD[1]**
**Demographics**			
Age, years	72.9±10.9	72.8±10.9	0.007
Female	1,384 (50.9)	1,320 (48.6)	0.047
Race			0.021
African American	397 (14.6)	396 (14.6)	
Asian	67 (2.5)	61 (2.2)	
Caucasian	2,005 (73.8)	2,000 (73.6)	
Other/unknown	249 (9.2)	261 (9.6)	
Medical insurance type			0.061
Commercial	513 (18.9)	526 (19.4)	
Medicaid	200 (7.4)	218 (8.0)	
Medicare	1,875 (69.0)	1,875 (69.0)	
Unknown	130 (4.8)	99 (3.6)	
**Clinical characteristics**			
BMI, kg/m^2	32.7±7.8	33.2±7.7	0.061
CKD stage			
Diagnosis code at index			0.000
Stage 3	2,022 (74.4%)	2,022 (74.4%)	
Stage 4	696 (25.6%)	696 (25.6%)	
eGFR closest to index[2], ml/min/1.73 m^2			0.000
Stage 3b (30 to <45)	1,960 (72.1)	1,960 (72.1)	
Stage 4 (15 to <30)	758 (27.9)	758 (27.9)	
Mean ± SD	33.8 ± 7.3	33.7 ± 7.4	0.001
Median (25%, 75% range)	34.8 (29.2, 39.6)	34.8 (28.8, 39.7)	
Hyperkalemia			
Total number of hyperkalemia events during baseline			
Mean ± SD	3.5 ± 2.1	0.0 ± 0.0	2.334
Median (25%, 75% range)	3.0 (2.0, 4.0)	0.0 (0.0, 0.0)	
Serum K+ lab concentration closest to index date, mmol/L			
Mean ± SD	5.1 ± 0.6	4.2 ± 0.4	1.697
Median (25%, 75% range)	5.1 (4.7, 5.4)	4.2 (3.9, 4.5)	
Hyperkalemia severity			1.649
≤5	1,152 (42.4)	2,718 (100.0)	
>5 to <5.5	919 (33.8)	0 (0.0)	
5.5 to <6	538 (19.8)	0 (0.0)	
≥6	109 (4.0)	0 (0.0)	
Most severe serum K+ concentration during baseline, mmol/L			
Mean ± SD	5.6 ± 0.4	4.4 ± 0.4	3.039
Median (25%, 75% range)	5.5 (5.3, 5.8)	4.5 (4.2, 4.7)	
Hyperkalemia severity			0.821
≤5	0 (0.0)	2,718 (100.0)	
>5 to <5.5	1,188 (43.7)	0 (0.0)	
5.5 to <6	1,163 (42.8)	0 (0.0)	
≥6	367 (13.5)	0 (0.0)	
Comorbidities			
Charlson comorbidity index	3.9±2.1	4.0±2.1	0.034
Hyperkalemia-related comorbidities			

Acute kidney injury	1,204 (44.3)	1,213 (44.6)	0.007
Coronary artery disease	1,153 (42.4)	1,235 (45.4)	0.061
Hypertension	2,572 (94.6)	2,580 (94.9)	0.013
Edema	943 (34.7)	938 (34.5)	0.004
Cardiovascular-related comorbidities			
Cerebrovascular disease	583 (21.4)	605 (22.3)	0.020
Congestive heart failure	1,062 (39.1)	1,083 (39.8)	0.016
Myocardial infarction	400 (14.7)	428 (15.7)	0.029
Peripheral vascular disease	926 (34.1)	880 (32.4)	0.036
**Medication use**			
Any RAASi	2,282 (84.0)	2,282 (84.0)	0.000
ACE inhibitor	1,361 (50.1)	1,137 (41.8)	0.166
ARB	937 (34.5)	1,140 (41.9)	0.154
ARN inhibitor	54 (2.0)	41 (1.5)	0.037
MRA	489 (18.0)	312 (11.5)	0.185
Beta blockers	1,754 (64.5)	1,822 (67.0)	0.053
**Healthcare resource utilization**			
Any inpatient visits	906 (33.3)	844 (31.1)	0.049
Any CKD-related inpatient visit	789 (29.0)	703 (25.9)	0.071
Any emergency department visits	851 (31.3)	870 (32.0)	0.015

Values are reported as mean±SD median (25%, 75% range), or n (%).

Abbreviations: ACE, angiotensin-converting enzyme; ARB, angiotensin receptor blocker; ARN, angiotensin receptor/neprilysin; BMI, body mass index; CKD, chronic kidney disease; eGFR, estimated glomerular filtration rate; MRA, mineralocorticoid receptor antagonist; RAASi, renin-angiotensin-aldosterone system inhibitor; SD, standard deviation; SMD, standardized mean difference.

Notes:

[1] SMD values >0.2 indicate an imbalance between cohorts.

[2] eGFR was assessed in the outpatient setting.

Supplemental Figure S1. Selection of patients with recurrent hyperkalemia (recurrent hyperkalemia sub-analysis) and the subset with mild hyperkalemia (mild and recurrent hyperkalemia sub-analysis).

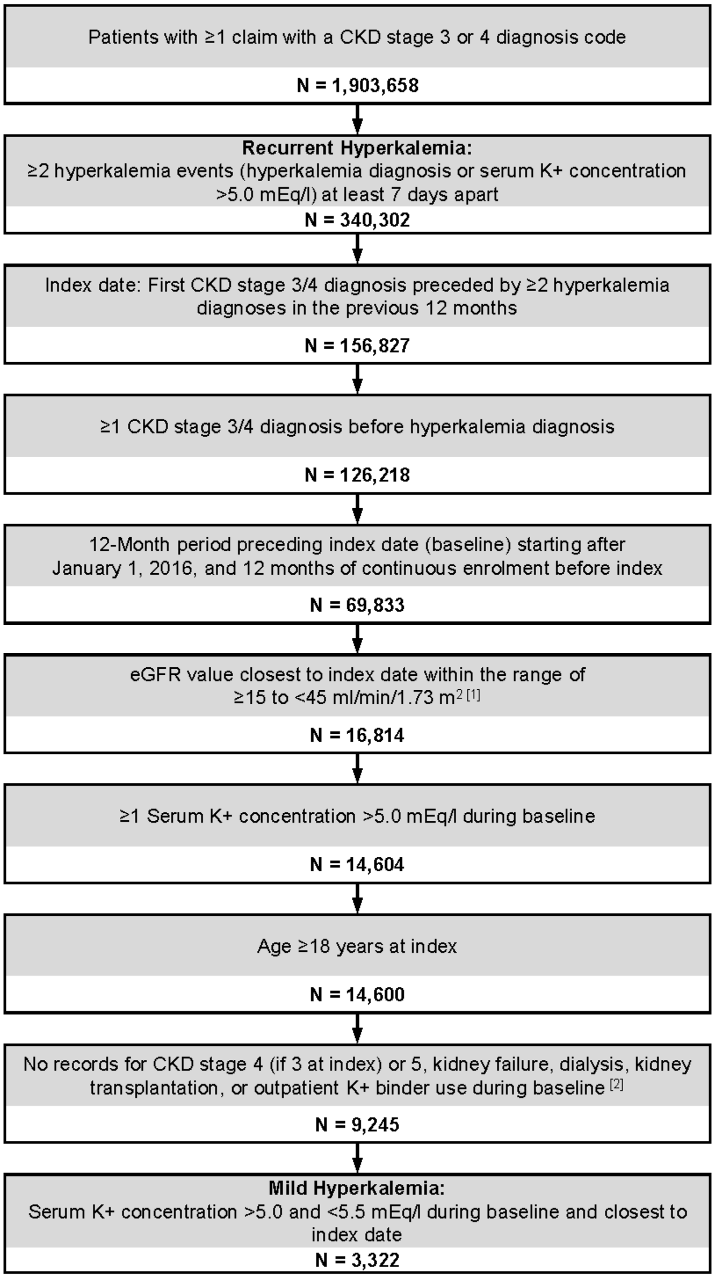

Abbreviations: CKD, chronic kidney disease; eGFR, estimated glomerular filtration rate; K+, potassium.

Notes:

[1] eGFR was assessed in the outpatient setting only, whereas serum K+ was assessed in any setting.

[2] Patients with advanced CKD stages (i.e., 4 [if stage 3 at index], 5, or kidney failure) during baseline were excluded based on diagnosis codes.

Supplemental Figure S2. Selection of patients in the sub-analyses

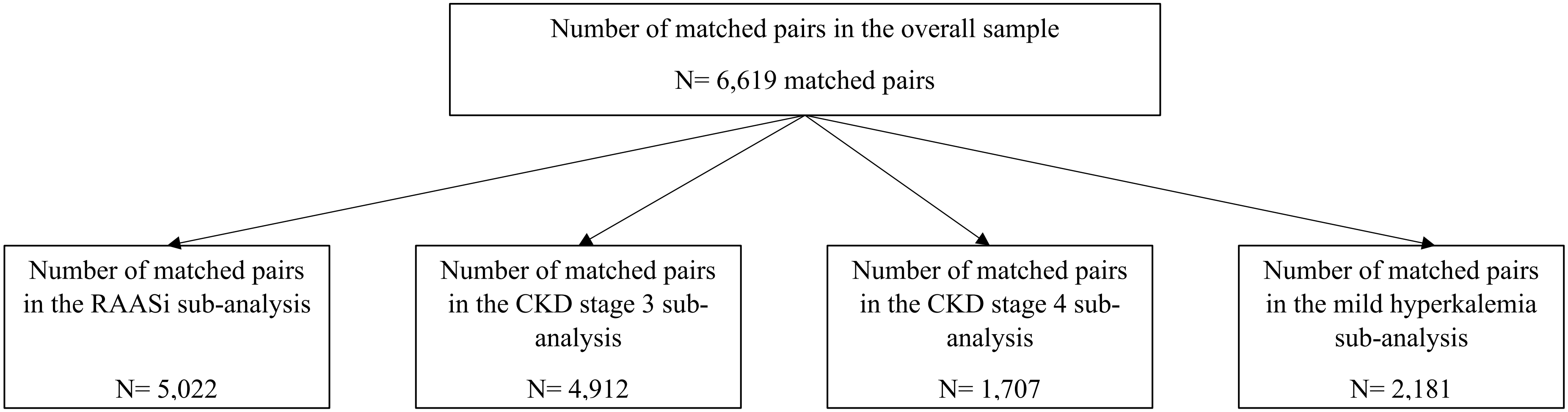

Note: The sub-analyses are not mutually exclusive.

Supplemental Figure S3. Forest plot of CKD progression hazard ratios in the type 2 diabetes mellitus, recurrent hyperkalemia, and mild and recurrent hyperkalemia sub-analyses.[1]

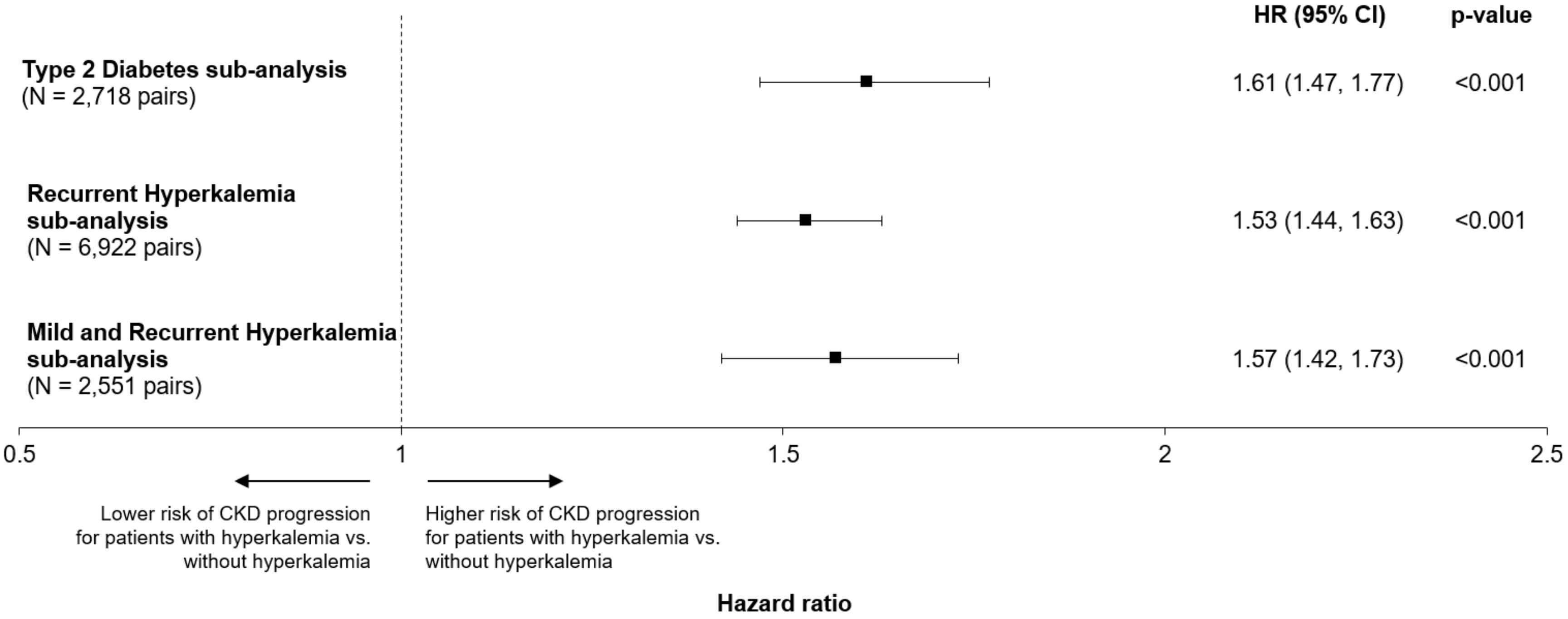

Abbreviations: CI, confidence interval; CKD, chronic kidney disease; HR, hazard ratio.

Note:

[1] CKD progression was defined as a diagnosis of CKD stage 4 (if stage 3 at index) or 5, kidney failure, dialysis, or kidney transplantation.

Supplemental Figure S4. Forest plot of secondary and exploratory CKD progression hazard ratios in the overall sample.

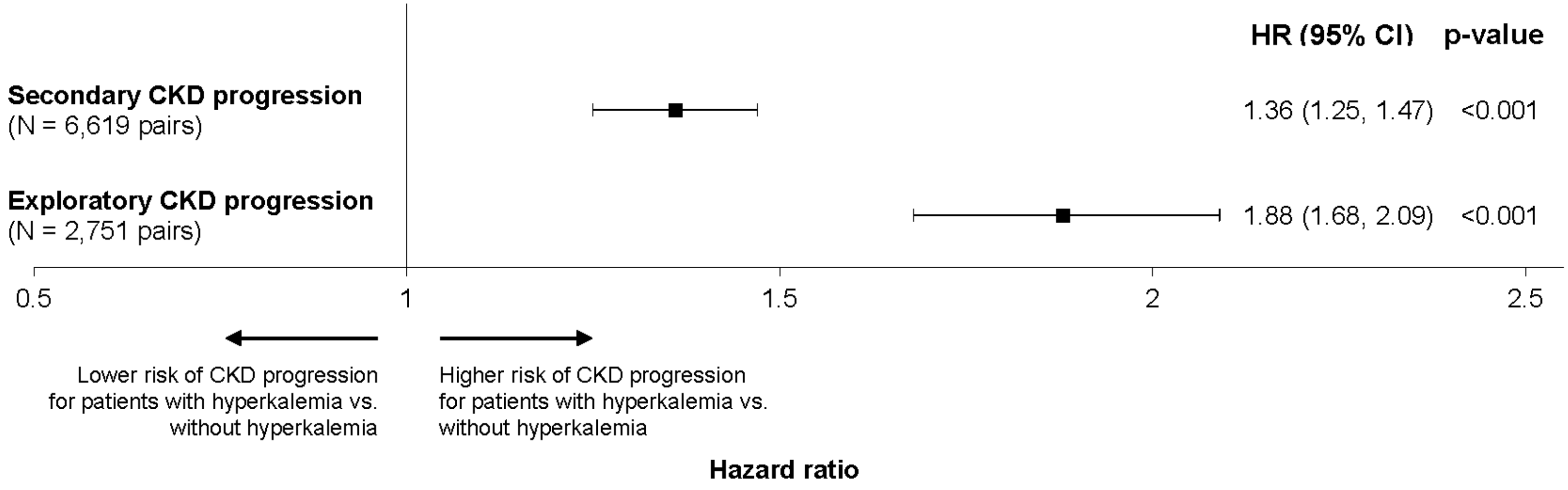

Abbreviations: CI, confidence interval; HR, hazard ratio.

Notes:

[1] Data was subset to matched pairs of 4,604 patients with ≥1 eGFR lab value recorded during the baseline period and at least 4 weeks after index during follow-up. A period of at least 4 weeks was chosen to ensure people had time to have a change in eGFR values.

[2] Secondary CKD progression was defined as patients with a diagnosis of stage 5 CKD or ESKD, dialysis, or kidney transplant.

[3] Exploratory CKD progression was defined as patients with a decline in eGFR from baseline of ≥30% at least 4 weeks after index, or dialysis or kidney transplant.

Supplemental Figure S5. Forest plot of mortality hazard ratios in the recurrent hyperkalemia and mild and recurrent hyperkalemia sub-analyses.

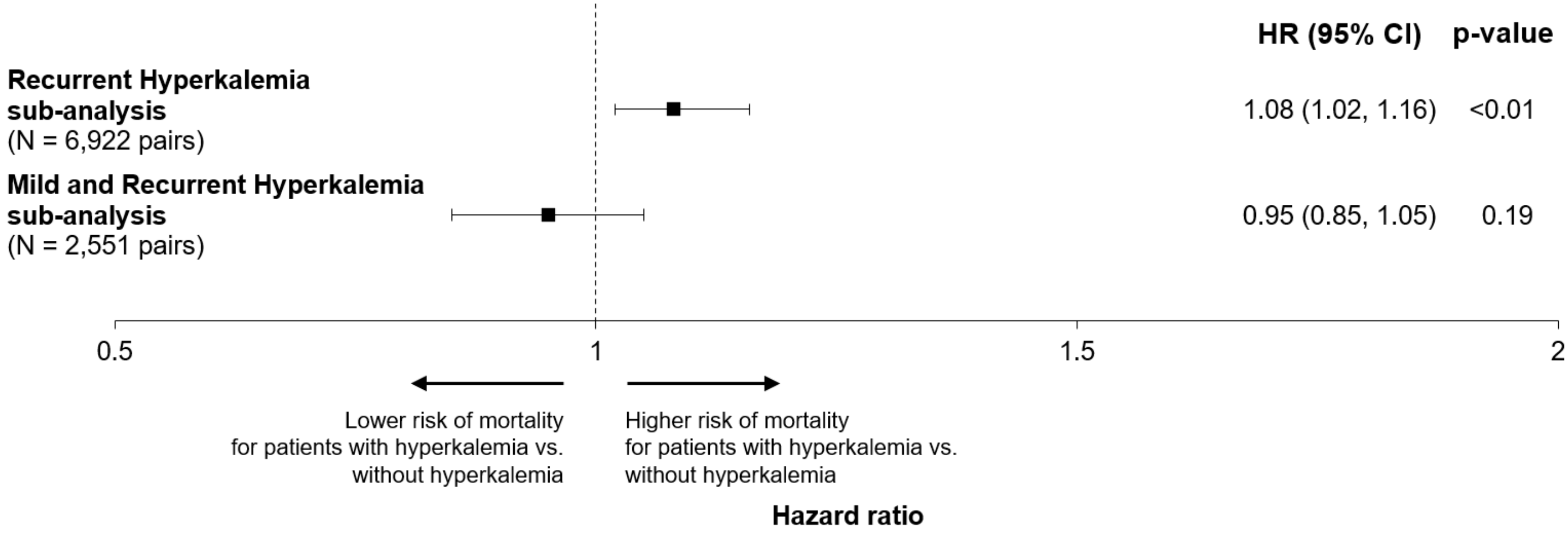

Abbreviations: CI, confidence interval; HR, hazard ratio.
